# Two Hearts in One Man

**Published:** 1898-12

**Authors:** 


### TWO HEARTS IN ONE MAN.

PLAINFIELD, N. J., November 9th.

William King, of New Bedford, Mass., has aroused the curiosity of the Plainfield medical fraternity as the most peculiar example on record of a man with two hearts. King is visiting his cousin, Thomas Martin, the jail warden in this city, and has been examined by Dr. Long, of the Muhlenburg Hospital staff. Dr. Long says King undoubtedly has two hearts. Both of them are capable of displacement and can be moved at will to different parts of the abdomen and separated one from the other, so that the beating in unison can be noted.

King is a colored man and claims to be one hundred years old and a veteran of the war of 1812. One of King's hearts is on the right and the other on the left side. By a muscular movement he can move one to the lower part of the abdomen without throwing it out of beat with the other.—*Philadelphia Times*, Nov. 10th.

